# Update in the Molecular Pathogenesis of Sebaceous Carcinoma

**DOI:** 10.3390/biology15191771

**Published:** 2026-10-06

**Authors:** Francisco Mano, João Teixeira, José Carlos Cardoso

**Affiliations:** 1 Department of Dermatology and Venereology, Coimbra University Hospital, Coimbra Local Health Unit, Praceta Professor Mota Pinto, Celas, 3004-561 Coimbra, Portugal; 2 Faculty of Medicine, University of Coimbra, Azinhaga de Santa Comba, Celas, 3000-548 Coimbra, Portugal

**Keywords:** sebaceous carcinoma, ocular sebaceous carcinoma, extraocular sebaceous carcinoma, mismatch repair deficiency, microsatellite instability, Lynch syndrome, Muir–Torre syndrome, *TP53*, *RB1*, *ZNF750*, *NOTCH1*, tumor mutational burden, immunotherapy

## Abstract

Sebaceous carcinoma is an uncommon but potentially aggressive adnexal malignancy whose molecular pathogenesis has been substantially clarified in recent years. Rather than representing a single molecular entity, sebaceous carcinoma is now understood as a biologically heterogeneous group of tumors arising through distinct yet sometimes overlapping oncogenic pathways. Recent genomic and multi-omics studies have identified several major molecular subsets, including mismatch repair-deficient (dMMR)/microsatellite instability (MSI)-associated tumors, ultraviolet (UV) radiation-driven tumors, and pauci-mutational tumors characterized predominantly by alterations in cell-cycle and epithelial differentiation regulators such as *TP53*, *RB1*, and *ZNF750*. These findings have also reinforced biologically relevant differences between ocular and extraocular sebaceous carcinoma, with periocular tumors more often enriched for *TP53*/*RB1*/*ZNF750*-driven mechanisms and extraocular tumors more frequently showing UV-related and dMMR signatures. In addition to these core pathways, recent evidence has highlighted the contribution of NOTCH signaling, phosphatidylinositol 3-kinase (PI3K)/protein kinase B (AKT)/mechanistic target of rapamycin (mTOR) pathway alterations, copy-number changes including *MYC* amplification, rare human papillomavirus (HPV)-associated cases, and emerging transcriptomic abnormalities involving cholesterol metabolism and sebaceous differentiation programs. These advances not only deepen current understanding of sebaceous carcinoma biology but also have important diagnostic, prognostic, and therapeutic implications, particularly in relation to Lynch/Muir–Torre syndrome screening, molecular subclassification, and the potential use of immune checkpoint inhibitors and other targeted strategies in selected patients. This review provides an updated overview of the molecular pathogenesis of sebaceous carcinoma, focusing on the main genomic, transcriptomic, and biologically actionable alterations described to date, and discusses their implications for tumor classification, pathogenesis, and future translational research.

## Simple Summary

Sebaceous carcinoma is a rare but potentially aggressive skin cancer that develops from oil-producing (sebaceous) glands, most often around the eyelids but sometimes elsewhere on the body. For many years it was treated as a single type of tumor, diagnosed mainly by its appearance under the microscope. Recent genetic studies have shown that sebaceous carcinoma is instead a group of biologically different tumors, each arising through a different combination of DNA changes, including defects in DNA-repair genes, sun-damage-related mutations, and disruption of genes that control cell growth and skin-cell maturation. This review brings together current knowledge on these mechanisms, explains how they differ between tumors arising near the eye and those arising elsewhere on the body, and discusses how this information can already help doctors identify patients who may carry an inherited cancer syndrome, refine difficult diagnoses, and, in the future, help select patients who might benefit from immune-based or targeted drug treatments. By clarifying these distinct disease routes, this work aims to support more precise diagnosis and more personalized care for patients with this rare cancer.

## 1. Introduction

Sebaceous carcinoma is a rare adnexal malignancy showing sebaceous differentiation and a broad clinicopathological spectrum. Although traditionally approached as a single diagnostic entity, it is increasingly recognized as a biologically heterogeneous tumor group with variable anatomic distribution, clinical behavior, syndromic associations, and molecular architecture. The distinction between ocular or periocular sebaceous carcinoma and extraocular sebaceous carcinoma is particularly relevant, as these tumors differ not only in clinical presentation and diagnostic challenges but also in their underlying molecular drivers.

Historically, sebaceous carcinoma has attracted interest because of its association with Muir–Torre syndrome, a phenotypic variant of Lynch syndrome characterized by sebaceous neoplasms and internal malignancies [1]. This association placed mismatch repair deficiency and microsatellite instability at the center of early molecular discussions. However, more recent genomic and multi-omics studies have substantially expanded this view, showing that only a subset of sebaceous carcinomas are driven by mismatch repair deficiency, while others arise through ultraviolet radiation-induced mutagenesis, alterations in cell-cycle regulators, epithelial differentiation pathways, copy-number changes, or rarer viral and polymerase-associated mechanisms.

A major conceptual advance was the recognition that sebaceous carcinoma can be divided into biologically distinct molecular classes. Early whole-exome sequencing studies identified pauci-mutational tumors, mismatch repair-deficient tumors, and ultraviolet (UV) radiation-driven tumors as major pathogenic categories [2]. Subsequent studies refined this framework and demonstrated that ocular and extraocular sebaceous carcinomas are not molecularly interchangeable. Periocular tumors appear more frequently enriched for alterations affecting *TP53*, *RB1*, and *ZNF750*, whereas extraocular tumors more often show ultraviolet mutational signatures, mismatch repair deficiency, higher tumor mutational burden, and alterations involving pathways such as NOTCH signaling.

Clinically, periocular sebaceous carcinoma typically presents as an eyelid nodule or diffuse thickening and may mimic chalazion or chronic blepharoconjunctivitis, whereas extraocular tumors usually present as nonspecific cutaneous nodules or plaques. Periocular disease has a greater reported propensity for nodal or distant metastasis, while population-based analyses of overall mortality have produced partly conflicting site-based estimates. Lynch/Muir-Torre association and UV-related carcinogenesis are more characteristic of extraocular disease. These epidemiological, clinical, prognostic, diagnostic, and therapeutic similarities and differences are summarized in Table 1. In this review, ocular sebaceous carcinoma denotes periocular/ocular-adnexal carcinoma arising on the eyelid; primary intraocular sebaceous carcinoma is a separate, exceptionally rare entity and is not considered here.

These molecular insights have practical implications. Identification of mismatch repair deficiency may prompt evaluation for Lynch/Muir–Torre syndrome and may support the use of immune checkpoint blockade in advanced disease. Recognition of ultraviolet-driven or highly mutated tumors may also be relevant for immunotherapeutic strategies, while detection of potentially actionable alterations in pathways such as PI3K/AKT/mTOR, receptor tyrosine kinase signaling, or DNA repair may guide individualized treatment in selected patients. In parallel, emerging transcriptomic and multi-omics studies have highlighted the importance of lipid metabolism, cholesterol homeostasis, and sebaceous differentiation programs, suggesting that sebaceous carcinoma should be understood not only as a tumor of genomic damage but also as a malignancy of aberrant lineage differentiation.

This review summarizes current knowledge on the molecular pathogenesis of sebaceous carcinoma, with emphasis on genomic, transcriptomic, and biologically actionable alterations. Particular attention is given to the distinction between ocular and extraocular disease, the role of mismatch repair deficiency and ultraviolet mutagenesis, and the translational implications of recent molecular discoveries for diagnosis, classification, prognostication, and future therapeutic development.

## 2. Literature Search Strategy

**Figure 1:**
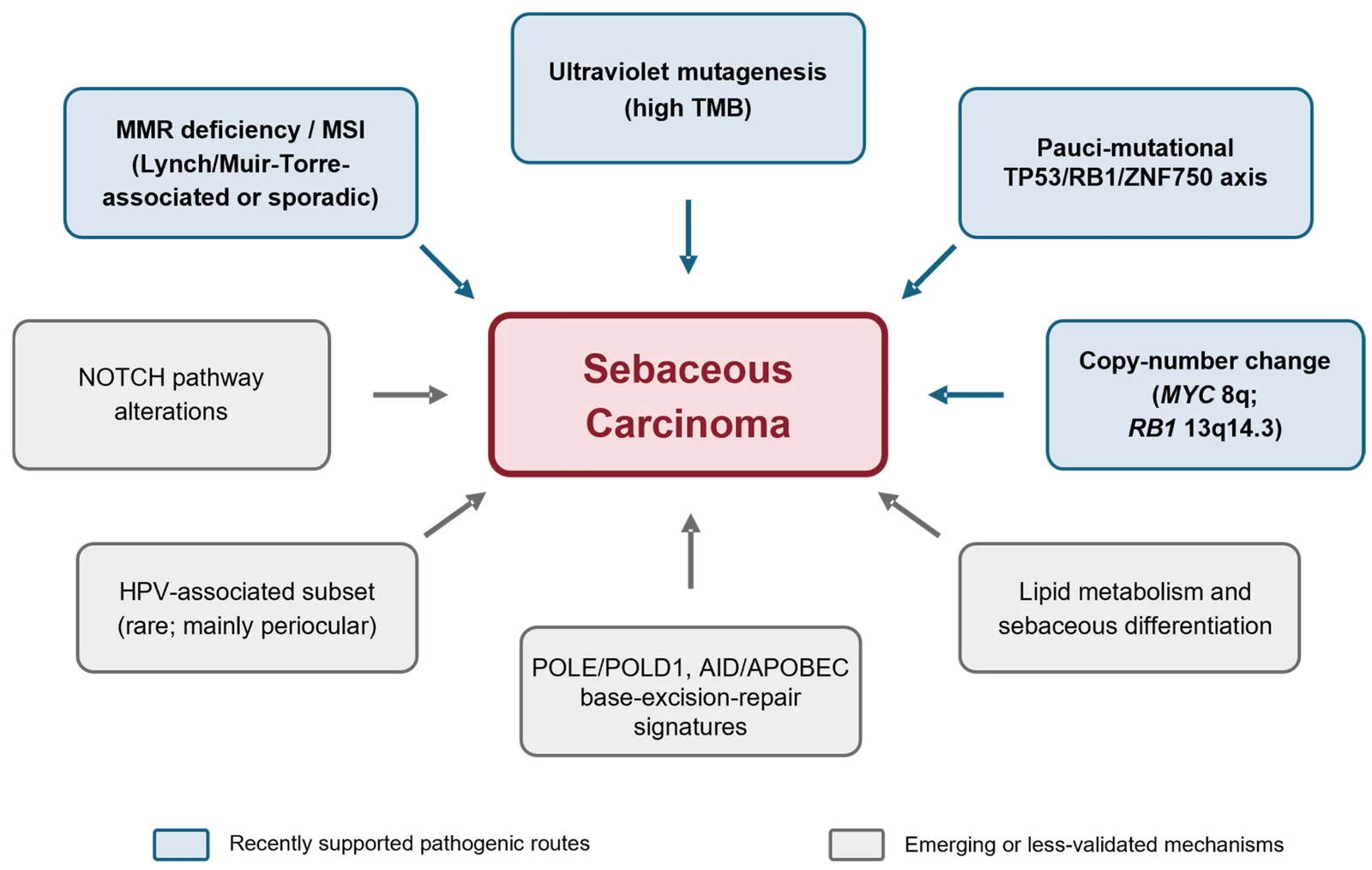
Integrated model of sebaceous carcinoma molecular pathogenesis. Blue boxes denote recurrently supported pathogenic routes, whereas grey boxes denote emerging or less-validated mechanisms. NOTCH alterations are recurrent but remain best interpreted as differentiation-associated findings rather than established therapeutic targets. The routes overlap and should not be regarded as mutually exclusive in every tumor. Blue arrows indicate recurrently supported routes, grey arrows indicate emerging or less-validated mechanisms, and the red central box indicates convergence on the sebaceous carcinoma phenotype. Abbreviations: AID, activation-induced cytidine deaminase; APOBEC, apolipoprotein B mRNA-editing enzyme catalytic polypeptide-like; HPV, human papillomavirus; MMR, mismatch repair; MSI, microsatellite instability; TMB, tumor mutational burden.

**Figure 2:**
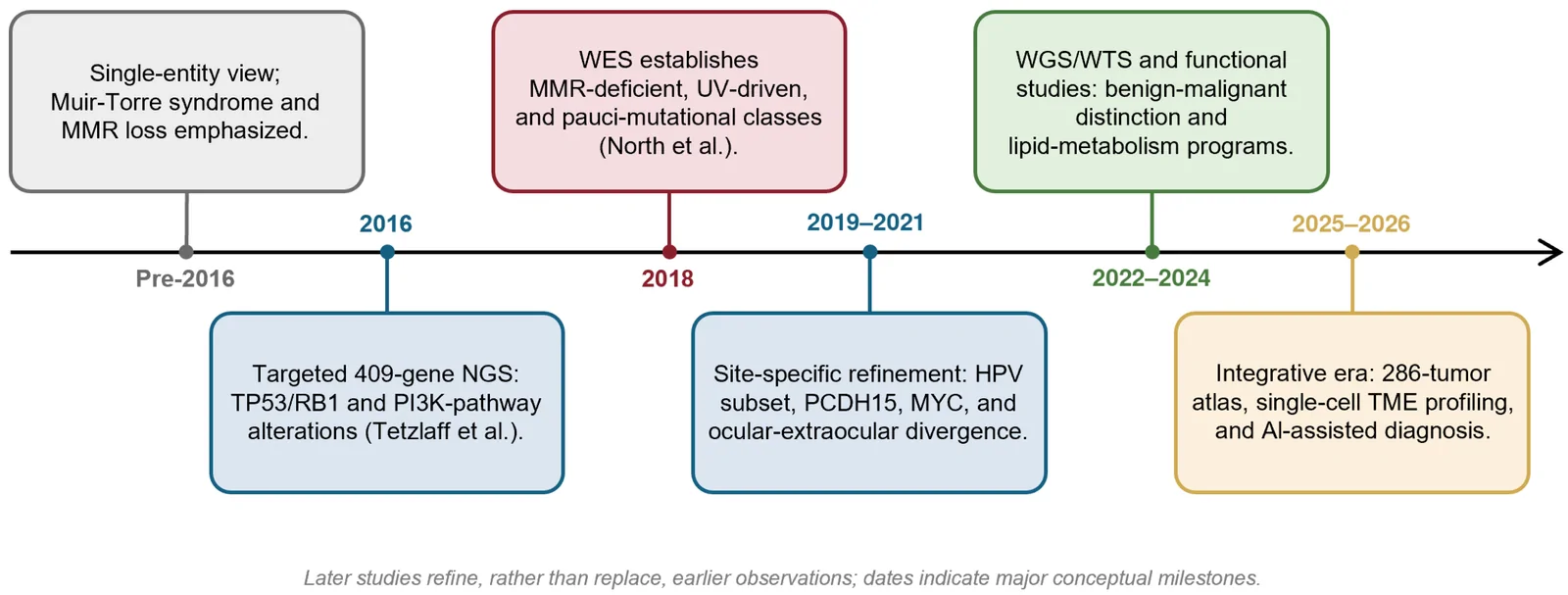
Evolution of understanding of sebaceous carcinoma molecular pathogenesis. Targeted sequencing in 2016 identified recurrent *TP53*/*RB1* and PI3K-pathway alterations [7]; whole-exome sequencing in 2018 established the MMR-deficient/MSI, UV-driven, and pauci-mutational framework [2]. Subsequent site-specific, whole-genome/transcriptome, single-cell, and artificial-intelligence-assisted studies progressively refined, rather than replaced, this model. The 2019–2021 site-specific phase is represented by genomic, transcriptomic, and HPV-focused studies [8,9,10,11,12,13,14,15]; the 2022–2024 whole-genome/transcriptome and functional phase by integrated molecular studies of lipid metabolism and benign–malignant distinction [14,16]; and the 2025–2026 integrative phase by the 286-tumor atlas, single-cell profiling, and AI-assisted diagnostic studies [5,17,18,19]. Abbreviations: AI, artificial intelligence; HPV, human papillomavirus; MMR, mismatch repair; MSI, microsatellite instability; NGS, next-generation sequencing; TME, tumor microenvironment; UV, ultraviolet; WES, whole-exome sequencing; WGS, whole-genome sequencing; WTS, whole-transcriptome sequencing.

**Figure 3:**
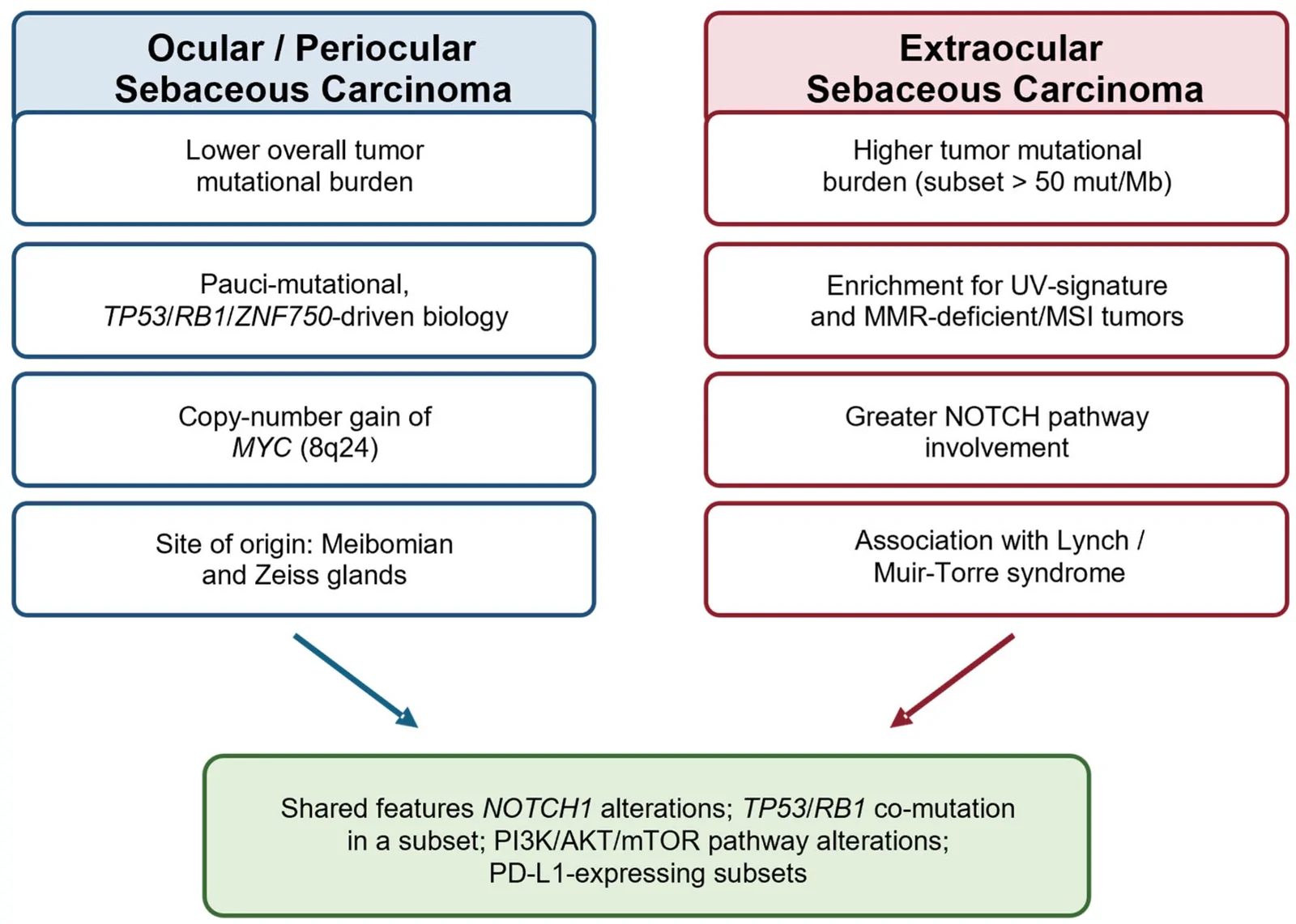
Molecular divergence between periocular (ocular-adnexal) and extraocular sebaceous carcinoma. Periocular tumors are more frequently enriched for pauci-mutational *TP53*/*RB1*/*ZNF750*-associated mechanisms and copy-number changes such as *MYC* gain, whereas extraocular tumors more often include mismatch repair-deficient, UV-driven, highly mutated, and NOTCH-altered subsets. The terms ocular and periocular are used for the same eyelid/ocular-adnexal category because the available molecular studies do not stratify them as separate groups. This category may include tumors involving the cutaneous and/or conjunctival aspect of the eyelid, but it excludes primary intraocular sebaceous carcinoma. Abbreviations: MMR, mismatch repair; MSI, microsatellite instability; PD-L1, programmed death-ligand 1; PI3K, phosphatidylinositol 3-kinase; TMB, tumor mutational burden; UV, ultraviolet.

This narrative review was based on a targeted literature search of PubMed/MEDLINE, complemented by relevant full-text articles and reference lists from primary studies and recent reviews. Searches were performed using combinations of the following terms: “sebaceous carcinoma”, “sebaceous gland carcinoma”, “ocular sebaceous carcinoma”, “extraocular sebaceous carcinoma”, “mismatch repair”, “microsatellite instability”, “Muir–Torre syndrome”, “Lynch syndrome”, “*TP53*”, “*RB1*”, “*ZNF750*”, “*NOTCH1*”, “*PIK3CA*”, “*MYC* amplification”, “HPV”, “transcriptome”, “microRNA”, “cholesterol metabolism”, “PD-L1”, “tumor mutational burden”, and “immunotherapy”. Priority was given to genomic, transcriptomic, multi-omics, immunohistochemical, and translational studies directly addressing sebaceous carcinoma biology. Because this was not designed as a systematic review, formal risk-of-bias assessment and meta-analysis were not performed. The search covered the literature published between January 2013 and August 2026, with the final search conducted on 6 August 2026. Titles and abstracts identified through these searches were screened for relevance to the molecular pathogenesis, classification, or translational management of sebaceous carcinoma; full texts were retrieved for records meeting these criteria, and reference lists of retrieved articles and recent reviews were hand-searched to identify additional eligible studies. Case reports without molecular data, non-English-language articles, and studies focusing exclusively on non-sebaceous adnexal tumors were not prioritized for inclusion. During the preparation of this manuscript, the authors used generative AI-assisted tools to assist with literature synthesis and restructuring of narrative text based on sources retrieved and verified by the authors and generation of the schematic diagrams in Figure 1, Figure 2 and Figure 3 from content and layout specifications provided by the authors. The authors critically reviewed, fact-checked against primary sources, and edited all AI-assisted content and take full responsibility for the accuracy, originality, and scientific integrity of the final manuscript. The PubMed/MEDLINE database is available at https://pubmed.ncbi.nlm.nih.gov/ (accessed on 6 August 2026).

## 3. Historical Framework: From a Single Entity to Molecular Classes

The molecular interpretation of sebaceous carcinoma changed substantially with the application of high-throughput sequencing. Earlier clinicopathological thinking often treated sebaceous carcinoma as a rare but relatively uniform adnexal malignancy, with Muir–Torre syndrome as the main molecular association. Whole-exome sequencing challenged this view by demonstrating that sebaceous carcinomas can arise through different mutational processes.

North and colleagues proposed a foundational three-class model of sebaceous carcinoma based on whole-exome sequencing [2]. In that study, tumors segregated into: (i) pauci-mutational tumors, including the ocular tumors in the cohort; (ii) mismatch repair (MMR)-deficient or microsatellite instability (MSI)-associated tumors, characterized by increased insertion/deletion burden and mismatch repair gene involvement; and (iii) ultraviolet radiation-driven tumors, characterized by high mutation burden and mutational signatures consistent with actinic damage. This model remains useful because it links mutational process, anatomic site, and clinicopathological phenotype.

The same framework also clarified why the association between sebaceous carcinoma and Muir–Torre syndrome, although clinically important, cannot explain the entire disease spectrum. Some tumors are mismatch repair-deficient and may reflect germline or somatic impairment of *MLH1*, *MSH2*, *MSH6*, or *PMS2*. Others are mismatch repair-proficient but highly mutated because of ultraviolet damage. A third group, particularly among ocular/periocular tumors, has comparatively low tumor mutational burden but recurrent disruption of cell-cycle and epithelial differentiation regulators.

More recent integrative studies have expanded this architecture. Rather than three rigid groups, sebaceous tumors appear to exist along a broader molecular continuum, with a major division between highly mutated tumors and pauci-mutated tumors. In a large worldwide multi-omics study of benign and malignant sebaceous tumors, high tumor mutational burden (TMB) was common across sebaceous tumors, except for many periocular carcinomas [5]. The most frequent processes associated with high mutational burden were mismatch repair deficiency, followed by ultraviolet-induced damage, *POLE*/*POLD1* mutations, and *AID*/*APOBEC*-associated signatures. Importantly, this study reinforced the role of biallelic *TP53* inactivation, often accompanied by *ZNF750* and/or *RB1* alterations, in sebaceous carcinoma [5].

Thus, the current molecular framework is best viewed as layered rather than categorical. Mismatch repair deficiency, ultraviolet mutagenesis, cell-cycle deregulation, epithelial differentiation defects, copy-number changes, viral oncogenesis, and transcriptomic reprogramming may each contribute to sebaceous carcinogenesis, with variable weight depending on anatomic site and tumor context. The major studies underpinning this molecular framework, together with their principal findings and translational relevance, are summarized in Table 2, the overall convergent pathogenic model is illustrated schematically in Figure 1, and the chronological evolution of these conceptual and methodological advances is summarized in Figure 2.

**Table 2:** Major molecular studies informing sebaceous carcinoma pathogenesis. Abbreviations: AR, androgen receptor; dMMR, mismatch repair-deficient; HPV, human papillomavirus; MMR, mismatch repair; MSI, microsatellite instability; NGS, next-generation sequencing; PD-L1, programmed death-ligand 1; SC-O, ocular/periocular sebaceous carcinoma; TMB, tumor mutational burden; UV, ultraviolet; WES, whole-exome sequencing.

Study	Design/Material	Main Molecular Findings	Translational Relevance
North et al., 2018 [2]	Whole-exome sequencing of sebaceous carcinoma	Three molecular classes: pauci-mutational, MSI/MMR-deficient, and UV-driven tumors	Foundational molecular classification; linked mutational process with anatomic site
Bao et al., 2019 [8]	Genomic analysis of ocular sebaceous carcinoma	*TP53*, *ZNF750*, and *RB1* typify ocular sebaceous carcinoma	Supports periocular cell-cycle/differentiation model
Tetzlaff et al., 2019 [13]	Targeted sequencing of ocular adnexal sebaceous carcinoma	HPV-driven and virus-negative tumors show nonoverlapping alterations	Defines rare HPV-associated subset
Xu et al., 2020 [11]	Whole-exome sequencing of ocular adnexal tumors	*TP53*, *ZNF750*, *RB1*, *NOTCH1*, and *PCDH15* recurrent; *PCDH15* associated with metastasis	Candidate prognostic marker for metastatic risk
Na et al., 2021 [10]	Targeted sequencing of ocular and extraocular tumors	*TP53*/*PIK3CA* frequent; *NOTCH1* more common in extraocular tumors; actionable variants identified	Supports ocular–extraocular divergence and molecular profiling in advanced disease
Peterson et al., 2021 [9]	NGS and copy-number analysis of ocular adnexal tumors	*TP53* and *RB1* common; *MYC* copy-number gain/amplification suggested	Highlights copy-number biology and *MYC* involvement
Saliba et al., 2021 [20]	PD-L1 immunohistochemical analysis	PD-L1 expression present in clinically relevant subset	Supports immunotherapy rationale
Wang et al., 2023 [14]	Integrated exome/transcriptome analysis	*SCARB1* increased and *PPARG* decreased; cholesterol metabolism dysregulated	Links tumor biology to sebaceous lipid metabolism
Starrett et al., 2024 [16]	Multi-omics profiling of sebaceous neoplasms	Molecular distinction of carcinoma from benign sebaceous tumors	Potential diagnostic classifiers and evolutionary insight
Ferreira et al., 2026 [5]	Worldwide multi-omics cartography of 286 sebaceous tumors	High TMB except many SC-O; dMMR, UV, *POLE*/*POLD1*, *APOBEC* signatures; *NOTCH1* frequent; *MYC*/8q, *RB1*/13q changes	Most comprehensive integrative framework to date
Zhang et al., 2025 [17]	Single-cell RNA profiling of ocular adnexal tumors	Complex tumor microenvironment and candidate biomarkers	Supports immune and stromal contribution
Byeon et al., 2025 [21]	Immunohistochemical prognostic study	Low nuclear YAP and low AR associated with higher local recurrence risk	Candidate prognostic biomarkers

## 4. Ocular Versus Extraocular Sebaceous Carcinoma: A Biologically Relevant Distinction

One of the most consistent findings across modern molecular studies is that ocular/periocular and extraocular sebaceous carcinomas are biologically distinct. This distinction is not absolute, but it is sufficiently reproducible to influence how the disease should be conceptualized, studied, and potentially treated.

Ocular sebaceous carcinoma most commonly arises in the eyelid region, where meibomian and Zeiss glands provide a rich sebaceous gland compartment. These tumors often show relatively low tumor mutational burden compared with extraocular lesions and are frequently enriched for alterations in *TP53*, *RB1*, and *ZNF750* [8,9]. In several studies, this profile supports a pauci-mutational, cell-cycle and differentiation-driven model of periocular tumorigenesis. Copy-number changes, including gains involving *MYC*, may also contribute to ocular sebaceous carcinoma biology [9].

Extraocular sebaceous carcinoma appears more heterogeneous. A proportion of cases shows mismatch repair deficiency and microsatellite instability, especially in the context of Lynch/Muir–Torre syndrome or acquired somatic mismatch repair alterations. Another subset shows ultraviolet mutational signatures and high tumor mutational burden, particularly in sun-exposed skin. These tumors may share molecular features with cutaneous squamous cell carcinoma, including enrichment for NOTCH pathway alterations and actinic mutational processes.

Targeted sequencing studies have also supported this anatomic divergence. Na and colleagues found *TP53* and *PIK3CA* mutations in both ocular and extraocular tumors, with slight predominance in ocular cases, whereas *NOTCH1* mutations were more common in extraocular tumors [10]. Clinically actionable variants involving tyrosine kinase genes, mismatch repair genes, and homologous recombination-related genes were also identified, suggesting that molecular testing may be particularly relevant in advanced or refractory cases [10].

This ocular–extraocular distinction should therefore be incorporated into the design of future studies. Combining ocular and extraocular tumors without stratification may obscure biologically meaningful associations, particularly for tumor mutational burden, mismatch repair status, UV signature, NOTCH pathway involvement, and copy-number events. These site-dependent molecular differences, together with the pathways shared between the two anatomic groups, are summarized schematically in Figure 3.

Because these molecular differences occur alongside broader epidemiological, clinical, and prognostic distinctions, Table 1 consolidates the features that differentiate periocular (ocular-adnexal) from extraocular sebaceous carcinoma, drawing on population-based surveillance data and molecular studies [1,3,5,6]. Separate molecular evidence for an ocular-versus-periocular subdivision is not currently available; consequently, they are retained as a single evidence-based category rather than presented as biologically distinct groups.

## 5. *TP53*, *RB1* and *ZNF750*: Cell-Cycle Control and Epithelial Differentiation

Among all recurrent molecular alterations described in sebaceous carcinoma, *TP53* and *RB1* constitute one of the most robust and repeatedly validated axes. *TP53* is a central regulator of DNA damage response, apoptosis, and genomic stability, while *RB1* controls G1/S cell-cycle progression through the retinoblastoma pathway. Disruption of both pathways provides a plausible mechanism for loss of proliferative control and malignant progression.

Peterson and colleagues used next-generation sequencing to evaluate ocular adnexal sebaceous carcinomas and found that the most common mutations occurred in *TP53* and *RB1* [9]. The authors concluded that alterations in these genes are among the commonest molecular events in sebaceous carcinoma. This finding is consistent with earlier and later studies showing frequent involvement of *TP53* and *RB1* in ocular/periocular sebaceous carcinoma [8,11].

*ZNF750* has emerged as a particularly informative gene because of its role in epithelial and keratinocytic differentiation. Loss-of-function alterations in *ZNF750* suggest that sebaceous carcinoma pathogenesis is not only a matter of cell-cycle deregulation, but also of impaired differentiation within a sebofollicular epithelial lineage. The recurrent involvement of *ZNF750* also helps explain why sebaceous carcinoma shares some molecular logic with squamous and epidermal differentiation programs, despite its distinctive sebaceous phenotype.

In the large integrative study by Ferreira and colleagues, biallelic *TP53* inactivation with concomitant *ZNF750* and/or *RB1* mutation was observed in sebaceous carcinoma, supporting the view that combined disruption of cell-cycle control and epithelial differentiation is a major pathogenic route [5]. The same study highlighted copy-neutral loss of heterozygosity as a mechanism that may contribute to biallelic inactivation of driver genes such as *TP53* and *ZNF750* [5].

Taken together, the *TP53*/*RB1*/*ZNF750* axis appears especially important in periocular and pauci-mutational sebaceous carcinoma. It defines a subset in which tumorigenesis may be less dependent on exogenous mutagenic burden and more dependent on disruption of intrinsic epithelial lineage and cell-cycle regulatory programs.

## 6. Mismatch Repair Deficiency, Microsatellite Instability and Lynch/Muir–Torre Syndrome

The relationship between sebaceous neoplasia and Lynch/Muir-Torre syndrome is one of the classic and most clinically important molecular associations in dermatopathology. Muir-Torre syndrome is generally regarded as a phenotypic variant of Lynch syndrome, which results from a germline pathogenic variant in *MLH1*, *MSH2*, *MSH6*, or *PMS2* followed by somatic inactivation of the remaining allele in a tumor. Muir-Torre syndrome is characterized by at least one Lynch-associated visceral malignancy and at least one cutaneous sebaceous neoplasm; mismatch repair deficiency is reported to be substantially less frequent in head-and-neck sebaceous carcinomas, a category that includes most periocular disease, than in tumors from other anatomic sites (55% versus 95% mismatch repair-deficient by immunohistochemistry in one series), suggesting a weaker Muir-Torre association for periocular disease, although this has not been quantified for the periocular subgroup specifically. Most Muir-Torre cases are related to *MSH2* (88–93%), with *MLH1* accounting for a smaller proportion (7–11%) [22]. Sebaceous adenoma, sebaceoma, or extraocular sebaceous carcinoma can therefore act as a visible marker of inherited cancer susceptibility and may precede detection of a visceral malignancy.

Mismatch repair deficiency leads to accumulation of replication errors, particularly in microsatellite regions, resulting in microsatellite instability and increased tumor mutational burden. In sebaceous carcinoma, mismatch repair deficiency may arise in the setting of germline Lynch syndrome, but it may also reflect somatic or acquired mismatch repair defects. Therefore, the presence of mismatch repair protein loss in a sebaceous tumor is clinically important but does not by itself establish germline Lynch syndrome.

Despite its limitations, immunohistochemical assessment of MLH1, PMS2, MSH2, and MSH6 is widely used to identify mismatch repair-deficient sebaceous neoplasms [12]. Abnormal immunohistochemistry should prompt careful clinical correlation, assessment of personal and family history of cancer, and consideration of genetic counseling and germline testing. Conversely, preserved mismatch repair protein expression does not necessarily exclude all hereditary risk, particularly when clinical suspicion is high.

From a pathogenic standpoint, mismatch repair-deficient sebaceous carcinomas form one of the major highly mutated subsets. In the original molecular classification, MSI/MMR-deficient tumors showed high insertion/deletion burden and frequent mismatch repair gene involvement [2]. In the more recent multi-omics cartography, mismatch repair deficiency emerged as the most frequent process associated with high tumor mutational burden among sebaceous tumors, exceeding ultraviolet damage as a global contributor in that dataset [5].

This pathway has translational relevance beyond hereditary cancer screening. Mismatch repair deficiency and microsatellite instability are tumor-agnostic predictive biomarkers for immune checkpoint blockade, providing a strong biological rationale in advanced sebaceous carcinoma. Nevertheless, sebaceous-carcinoma-specific treatment evidence remains limited, and one immunohistological cohort found no difference in immune-cell composition according to MMR status. In advanced, unresectable, recurrent, or metastatic disease, MMR/MSI assessment may therefore be considered as part of an individualized molecular workup rather than interpreted as a stand-alone predictor of response.

## 7. Ultraviolet-Driven Sebaceous Carcinoma and Overlap with Squamous Carcinogenesis

Ultraviolet radiation is another major mutational process in sebaceous carcinoma, especially in extraocular tumors arising on sun-exposed skin. UV-driven tumors typically show high tumor mutational burden and characteristic mutational signatures associated with actinic damage. This pathway places a subset of sebaceous carcinomas within the broader landscape of keratinocyte cancers driven by chronic ultraviolet exposure.

In the classification proposed by North and colleagues, UV-signature tumors represented a distinct molecular class, separate from MSI/MMR-deficient and pauci-mutational tumors [2]. These tumors displayed high mutation burden and were more often extraocular. Histologically, UV-driven tumors were associated with more infiltrative and less differentiated features in the original study, supporting a possible relationship between actinic mutational load and aggressive morphology.

The UV-driven subset also helps explain molecular overlap between extraocular sebaceous carcinoma and cutaneous squamous cell carcinoma. Both can arise on chronically sun-damaged skin and both may show high mutational burden, tumor suppressor disruption, and NOTCH pathway alterations. In contrast, basal cell carcinoma-like sonic hedgehog pathway dominance does not appear to define most sebaceous carcinomas, although hedgehog signaling may still contribute in selected periocular tumors.

Recognition of UV-driven sebaceous carcinoma has potential clinical relevance. High tumor mutational burden may increase immunogenicity and could partly explain why some advanced sebaceous carcinomas may respond to immune checkpoint inhibition even outside classical Lynch/Muir–Torre syndrome. However, tumor mutational burden alone should not be interpreted as a definitive predictive biomarker in sebaceous carcinoma without broader clinical and molecular correlation.

## 8. NOTCH Signaling and Differentiation-Associated Pathways

NOTCH signaling is one of the most recurrently reported pathways in sebaceous carcinoma, although, as with several other pathways discussed in this review, the supporting evidence comes mainly from moderate-sized cohorts and should be interpreted as an emerging association rather than an established mechanism. In squamous and adnexal epithelial tumors, NOTCH can act as a context-dependent regulator of differentiation and tumor suppression, and loss or mutation of NOTCH pathway components may impair epithelial maturation and facilitate malignant transformation.

Several studies suggest that *NOTCH1* alterations are particularly relevant in extraocular and highly mutated sebaceous carcinoma. Na and colleagues found *NOTCH1* mutations to be more common in extraocular sebaceous carcinoma, while pathway analysis suggested NOTCH signaling as a distinctive extraocular feature [10]. The large multi-omics study by Ferreira and colleagues further emphasized this pathway by identifying *NOTCH1* as the most frequently mutated gene across the global sebaceous tumor cohort [5].

The role of NOTCH in ocular sebaceous carcinoma is more complex. Whole-exome sequencing of ocular adnexal sebaceous carcinoma identified *NOTCH1* among recurrently mutated genes, and NOTCH pathway alterations consistently co-occurred with *TP53* and/or *RB1* mutations [11]. Xu and colleagues reported that *ZNF750* or NOTCH pathway gene mutations were present in a large proportion of ocular adnexal cases, with a trend toward mutual exclusivity between *ZNF750* and *NOTCH1* mutations [11]. This suggests that both pathways may converge on epithelial differentiation programs, even if their relative contribution varies by tumor subset.

At present, NOTCH alterations are best interpreted as markers of epithelial differentiation dysregulation rather than immediately actionable therapeutic targets in routine sebaceous carcinoma care. Nevertheless, their recurrence supports a broader model in which sebaceous carcinoma arises through disruption of lineage specification, keratinocytic/sebaceous differentiation, and cell-cycle control.

## 9. Phosphatidylinositol 3-Kinase (PI3K)/Protein Kinase B (AKT)/Mechanistic Target of Rapamycin (mTOR) Pathway and Other Potentially Actionable Alterations

Beyond the dominant axes of mismatch repair deficiency, ultraviolet damage, *TP53*/*RB1*/*ZNF750*, and NOTCH signaling, several studies have identified potentially actionable alterations in sebaceous carcinoma. These findings are particularly relevant because advanced sebaceous carcinoma is rare and lacks standardized systemic treatment strategies.

Alterations in the PI3K/AKT/mTOR pathway have been reported in small-to-moderate cohorts, particularly in ocular-adnexal sebaceous carcinoma, and should currently be regarded as recurrent findings rather than validated therapeutic targets. A 409-gene sequencing study of 27 tumors reported alterations converging on PI3K signaling, while Na and colleagues identified recurrent *PIK3CA* mutations and pathway enrichment in ocular tumors [7,10]. These observations justify further investigation and possible clinical-trial consideration, but they do not establish routine pathway-directed therapy.

Other potentially actionable genes have been reported across small cohorts, including *EGFR*, *ERBB2*, *ROS1*, *NF1*, and genes involved in DNA repair or homologous recombination. Na and colleagues identified variants involving tyrosine kinase genes and homologous recombination deficiency-associated genes [10], while Peterson and colleagues detected additional mutations in genes such as *NF1*, *PMS2*, *ROS1*, *KMT2C*, *NOTCH1*, *PCLO*, and *PTPRT* [9]. These observations support the use of broad molecular profiling in selected patients with unresectable, recurrent, metastatic, or treatment-refractory disease.

Nevertheless, caution is required. Many reported alterations occur in small series, and their biological relevance may vary according to whether they are true drivers, passenger mutations in highly mutated tumors, or variants of uncertain significance. At present, actionable molecular findings in sebaceous carcinoma should be interpreted within a multidisciplinary molecular tumor board whenever possible.

## 10. Copy-Number Alterations and *MYC* Amplification

Copy-number alterations are increasingly recognized as part of sebaceous carcinoma biology. Although early sequencing studies emphasized single-nucleotide variants and indels, subsequent work has shown that gains, amplifications, deletions, and copy-neutral loss of heterozygosity may contribute to tumorigenesis and progression.

*MYC* is the most notable copy-number-related oncogene in ocular sebaceous carcinoma. Peterson and colleagues identified low-level copy-number gain suggestive of *MYC* amplification in ocular adnexal sebaceous carcinoma and confirmed *MYC* copy-number gain using fluorescence in situ hybridization in selected cases [9]. MYC protein expression was present in most cases assessed by immunohistochemistry, suggesting that *MYC* may contribute to tumor biology even when amplification is not overt [9].

The large integrative study by Ferreira and colleagues further supported the relevance of copy-number changes. Amplification of 8q, including *MYC*, was associated with periocular sebaceous carcinoma, whereas amplification of 1q21.3 and chromosome 20 were shared by periocular and extraocular tumors [5]. Deletion of 13q14.3, where *RB1* resides, was also recurrent [5]. These observations provide a copy-number counterpart to the *TP53*/*RB1*/*ZNF750* mutational axis and suggest that dosage effects may be particularly important in ocular sebaceous carcinoma.

Copy-neutral loss of heterozygosity is also relevant because it can convert a heterozygous driver alteration into biallelic inactivation without changing total copy number. This mechanism may be important for tumor suppressor genes such as *TP53*, *ZNF750*, *MSH2*, *MLH1*, and *NOTCH1*. Future studies integrating mutation, copy number, methylation, and expression data will be essential to clarify which copy-number alterations are true drivers and which are secondary consequences of genomic instability.

## 11. HPV-Associated Sebaceous Carcinoma: A Rare Alternative Pathway

The role of human papillomavirus (HPV) in sebaceous carcinoma has historically been controversial. Current evidence supports a balanced interpretation: HPV is not a universal or dominant driver of sebaceous carcinoma, but it may define a rare molecular subset, particularly among ocular adnexal tumors.

Tetzlaff and colleagues described distinct biological types of ocular adnexal sebaceous carcinoma, separating HPV-driven tumors from virus-negative tumors [13]. These groups appeared to arise through non-overlapping molecular-genetic alterations. In that study, HPV-driven tumors tended to occur in younger patients and showed features distinct from the classic virus-negative *TP53*/*RB1*-associated pathway.

The recent multi-omics cartography of sebaceous tumors also supports the idea that viral oncogenesis exists but is uncommon [5]. HPV-associated cases should therefore be regarded as a minor pathogenic route rather than a general explanation for sebaceous carcinoma. From a practical standpoint, HPV testing may be considered in selected cases, particularly younger patients or tumors with morphology/immunophenotype suggestive of virus-associated biology, but it is not currently a universal requirement in sebaceous carcinoma evaluation.

This rare subset is conceptually important because it reinforces the broader theme of molecular heterogeneity. Sebaceous carcinoma can converge morphologically from distinct etiologic routes: mismatch repair deficiency, UV damage, cell-cycle/differentiation disruption, and, rarely, viral oncogenesis.

## 12. Transcriptomics, Lipid Metabolism and Sebaceous Differentiation

Genomic studies have provided the backbone of current molecular classification, but transcriptomic and multi-omics approaches are beginning to reveal additional layers of sebaceous carcinoma biology. These studies are especially important because sebaceous carcinoma is defined by lineage differentiation, and DNA sequencing alone may not fully capture aberrant sebaceous programming.

Integrated whole-exome and transcriptome sequencing of eyelid sebaceous carcinoma has suggested dysregulation of cholesterol metabolism. Wang and colleagues reported increased *SCARB1* and decreased *PPARG* expression, supporting the hypothesis that altered lipid handling and cholesterol homeostasis may participate in sebaceous carcinogenesis [14]. This is biologically plausible because normal sebaceous glands are highly specialized lipid-producing structures, and malignant transformation may involve disruption of the transcriptional programs that regulate sebocyte differentiation and lipid metabolism.

Multi-omics profiling has further supported the value of transcriptomic markers for distinguishing sebaceous carcinoma from benign sebaceous tumors. Starrett and colleagues used genomic and transcriptomic profiling to differentiate sebaceous carcinoma from benign sebaceous neoplasms and to provide insight into the genetic evolution of sebaceous carcinogenesis [16]. Such work suggests that future diagnostic classifiers may combine mutational signatures, copy-number profiles, and expression-based biomarkers.

MicroRNA studies also support a role for post-transcriptional regulation. Bladen and colleagues identified altered microRNAs in periocular sebaceous gland carcinoma, including hsa-miR-34a-5p and hsa-miR-16-5p, with differential expression of targets such as *BCL2* and *MYC* [15]. They also reported subtype-specific microRNA patterns associated with nodular versus pagetoid growth, suggesting that invasion and intraepithelial spread may be partly governed by non-coding RNA networks.

These observations broaden the conceptual model of sebaceous carcinoma. The disease should not be viewed only as a tumor of mutational damage. It is also a tumor of disturbed lineage identity, sebaceous differentiation, lipid metabolism, and transcriptional/post-transcriptional regulation.

## 13. Hedgehog, Hippo–Yes-Associated Protein (YAP) and Emerging Signaling Pathways

Several additional signaling pathways have been investigated in sebaceous carcinoma, although most remain less validated than *TP53*/*RB1*/*ZNF750*, mismatch repair deficiency, ultraviolet damage, and NOTCH signaling.

Hedgehog signaling has been studied in periocular sebaceous carcinoma. Bladen and colleagues reported evidence of hedgehog pathway activation compared with controls, suggesting that this pathway may participate in tumor biology [23]. However, hedgehog signaling does not appear to dominate sebaceous carcinoma in the way it dominates basal cell carcinoma. Its role is therefore best interpreted as contributory or context-dependent rather than defining.

The Hippo–YAP pathway has recently attracted interest as a possible prognostic axis in eyelid sebaceous carcinoma. YAP is a transcriptional coactivator regulated by the Hippo pathway and has context-dependent oncogenic or tumor-suppressive roles. In a single-institution retrospective cohort of 47 patients with eyelid sebaceous carcinoma, Byeon and colleagues found that low nuclear YAP expression was associated with higher local recurrence risk, and lower androgen receptor expression was associated with worse local recurrence-free survival [21]. These findings are hypothesis-generating rather than established: they derive from a single moderate-sized cohort, and YAP and androgen receptor expression should be regarded as candidate, not validated, prognostic markers until replicated in independent series.

Other emerging pathways include base-excision repair signatures and apolipoprotein B mRNA-editing enzyme catalytic polypeptide-like (*APOBEC*)-associated mutagenesis. Sangiorgi and colleagues reported a base-excision repair mutational signature in two eyelid sebaceous carcinomas, while the large multi-omics cartography identified *activation-induced cytidine deaminase (AID)*/*APOBEC* activation signatures among processes contributing to high tumor mutational burden [5,24]. These rare or newly described processes underscore that sebaceous carcinoma may arise through a wider range of DNA damage and repair abnormalities than previously appreciated.

At present, these emerging pathways should be considered hypothesis-generating. Their main value is to identify new biomarkers, refine molecular taxonomy, and suggest potential therapeutic vulnerabilities for future investigation.

## 14. Tumor Microenvironment, Programmed Death-Ligand 1 (PD-L1) Expression and Immunotherapy Rationale

The immunobiology of sebaceous carcinoma has gained importance as molecular studies have demonstrated the presence of mismatch repair-deficient, microsatellite instability-high, and tumor mutational burden-high subsets. These features are associated with increased neoantigen load and provide a biological rationale for immune checkpoint inhibition in selected patients.

Immune modulation in sebaceous carcinoma extends well beyond PD-L1 itself. In a series of 173 resected tumors, Saliba and colleagues characterized both tumoral and immune-infiltrating PD-L1 expression alongside CD8+ T-lymphocyte density: higher combined positive scores correlated with greater CD8+ infiltration, tumor-associated T-cell infiltrate was concentrated at the tumor periphery rather than the core, and PD-L1 expression (PD-L1+Tu, PD-L1+Inf, and CPS) was higher in extraocular and larger tumors, although CD8+ density itself did not correlate with anatomic location or lesion size [20]. This peripheral, extraocular-enriched pattern suggests that extraocular tumors may sustain a more active, if incompletely effective, cytotoxic T-cell response that the tumor counters through PD-L1-mediated checkpoint engagement, whereas periocular/pauci-mutational tumors may rely comparatively more on intrinsic proliferative and differentiation-pathway disruption than on active immune suppression. PD-L1 expression should not be interpreted in isolation, but it may contribute to a broader immunotherapy biomarker panel together with mismatch repair status, microsatellite instability, tumor mutational burden, and immune infiltrate.

Single-cell RNA profiling has further resolved the composition of this tumor microenvironment in ocular adnexal sebaceous carcinoma. Zhang and colleagues profiled 29,219 cells from primary and pagetoid-spread tumors and normal tarsal tissue, identifying an immune compartment comprising CD4+ T cells, regulatory T (Treg) cells, CD8+ T cells (including exhausted subpopulations), natural killer (NK) cells, naïve B cells, plasma cells, dendritic cells, and mast cells, alongside a stromal compartment of fibroblasts, endothelial cells, and smooth muscle [17]. The presence of regulatory T cells and exhausted CD8+ populations points to active local immunosuppression layered on top of PD-L1-mediated checkpoint engagement, while tumor cells themselves showed upregulated mitotic and oxidative-phosphorylation pathways and downregulated lipid biosynthesis and metabolism relative to normal tissue, and cells undergoing pagetoid spread specifically upregulated epithelial migration pathways [17]. This immune and stromal complexity, layered on tumor-intrinsic metabolic reprogramming, reinforces the idea that tumor behavior is shaped jointly by tumor-intrinsic mutations and microenvironmental interactions, and that the balance of immunosuppressive (Treg, exhausted CD8+) versus cytotoxic (active CD8+, NK) populations may itself differ across anatomic and molecular subsets, though dedicated studies stratifying the immune microenvironment by the mismatch repair-deficient, UV-driven, and pauci-mutational classes described earlier in this review have not yet been reported and represent an important gap for future work.

Clinically, immune checkpoint inhibitors may be most rational in mismatch repair-deficient/MSI-high tumors and possibly in UV-driven or tumor mutational burden-high tumors. However, evidence in sebaceous carcinoma remains limited, and prospective trials are difficult because of the rarity of the disease. Basket trials and molecularly selected case series may therefore be particularly important for defining the role of immunotherapy in advanced sebaceous carcinoma.

Beyond lymphocytic infiltration and PD-L1, myeloid remodeling may accompany malignant progression. Frouin and colleagues analyzed 123 sebaceous neoplasms and found greater infiltration by CD68-positive macrophages, CD163-positive M2 macrophages, and CD11c-positive dendritic cells in carcinomas than in benign sebaceous tumors. Immune-cell PD-L1 expression was also higher in carcinomas, but immune-cell composition did not differ according to MMR status or Muir-Torre syndrome [25]. These findings provide direct evidence of a carcinoma-associated myeloid microenvironment while cautioning against assuming that dMMR alone defines a distinct cellular infiltrate.

In a separate cohort of 52 eyelid sebaceous carcinomas, Singh and colleagues evaluated programmed cell death protein 1 (PD-1), PD-L1, cytotoxic T-lymphocyte-associated protein 4 (CTLA-4), CD4, and CD8 across tumor and stromal compartments. Tumor-infiltrating lymphocyte PD-1 expression was independently associated with poorer disease-free survival, whereas the prognostic associations of PD-L1 varied according to its tumor or stromal localization [26]. These single-cohort observations are relevant to immune escape and prognosis but have not been validated as predictive biomarkers of treatment response.

Available evidence therefore supports substantial immune heterogeneity, but it does not yet establish a validated immune classification aligned with the MMR-deficient, UV-driven, and pauci-mutational molecular classes. Table 3 distinguishes direct sebaceous-carcinoma observations from biologically plausible but untested subtype-specific adaptations.

## 15. Molecular Progression and Metastasis

**Figure 4:**
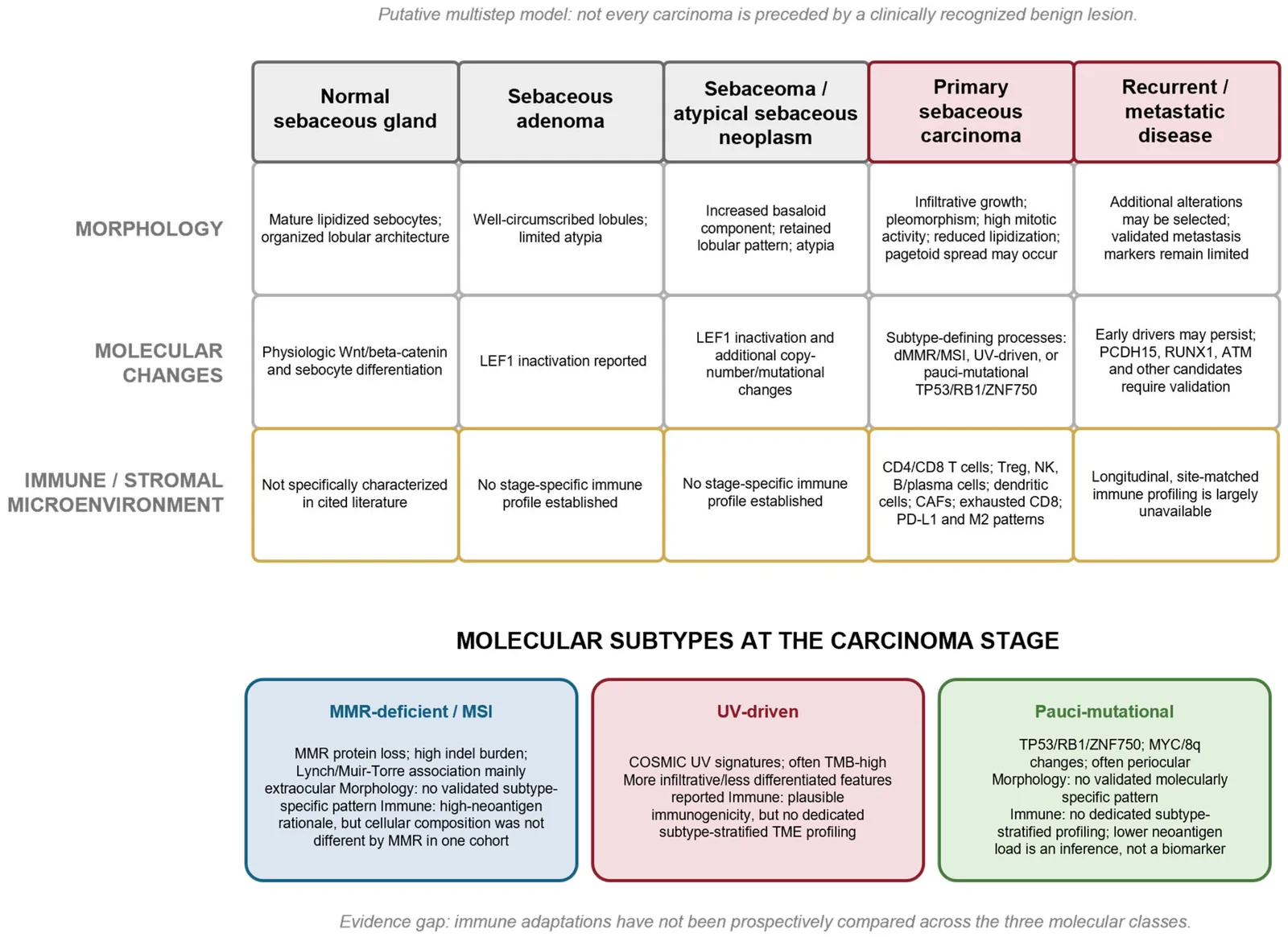
Proposed morphological, molecular, and immune evolution of sebaceous carcinoma. Multi-omics data support a putative precursor spectrum involving sebaceoma and atypical sebaceous neoplasms, but not every carcinoma is known to traverse a clinically recognized benign stage [16]. At the carcinoma stage, MMR-deficient/MSI, UV-driven, and pauci-mutational processes show distinct genomic features, whereas subtype-specific immune and stromal adaptations remain insufficiently characterized. Candidate progression-associated alterations require independent validation, and the figure explicitly separates direct observations from evidence gaps. Grey boxes depict normal or benign stages; pink boxes depict primary and recurrent/metastatic carcinoma; amber borders identify immune/stromal microenvironment features; and the blue, red, and green boxes denote the MMR-deficient/MSI, UV-driven, and pauci-mutational classes, respectively. Abbreviations: CAF, cancer-associated fibroblast; COSMIC, Catalogue of Somatic Mutations in Cancer; MMR, mismatch repair; MSI, microsatellite instability; NK, natural killer; PD-L1, programmed death-ligand 1; TMB, tumor mutational burden; TME, tumor microenvironment; Treg, regulatory T cell; UV, ultraviolet.

Figure 4 summarizes the morphological, molecular, and microenvironmental progression from normal sebaceous gland through adenoma and sebaceoma to carcinoma described throughout this review, including the point at which tumors diverge into the three molecular subtypes discussed above. Beyond this progression to a primary carcinoma, the molecular basis of recurrence and metastasis in sebaceous carcinoma remains incompletely understood. Most available studies include small numbers of metastatic cases, and the rarity of the disease limits statistically robust conclusions. Nevertheless, several observations are noteworthy.

Whole-exome sequencing of ocular adnexal sebaceous carcinoma identified recurrent alterations in *TP53*, *ZNF750*, *RB1*, *NOTCH1*, and *PCDH15* [11]. Xu and colleagues reported that *PCDH15* mutations were significantly associated with metastasis, with most *PCDH15*-mutated cases occurring in patients with nodal or distant metastatic disease [11]. Although this finding requires validation, it suggests that specific mutations may eventually help identify patients at higher metastatic risk.

Comparative analyses of primary and recurrent or metastatic lesions have also suggested clonal continuity in some cases. Persistence of mutations such as *TP53* or *PIK3CA* from primary tumor to recurrence/metastasis supports the concept that early driver events may be retained during progression, while additional alterations may accumulate under selective pressure.

The molecular determinants of local recurrence, pagetoid spread, lymphovascular invasion, and distant metastasis are likely to differ. Future studies should integrate detailed clinicopathological annotation with spatial sampling, longitudinal sequencing, and microenvironmental analysis to distinguish drivers of primary tumor initiation from drivers of progression.

## 16. Translational Implications for Diagnosis and Clinical Management

**Figure 5:**
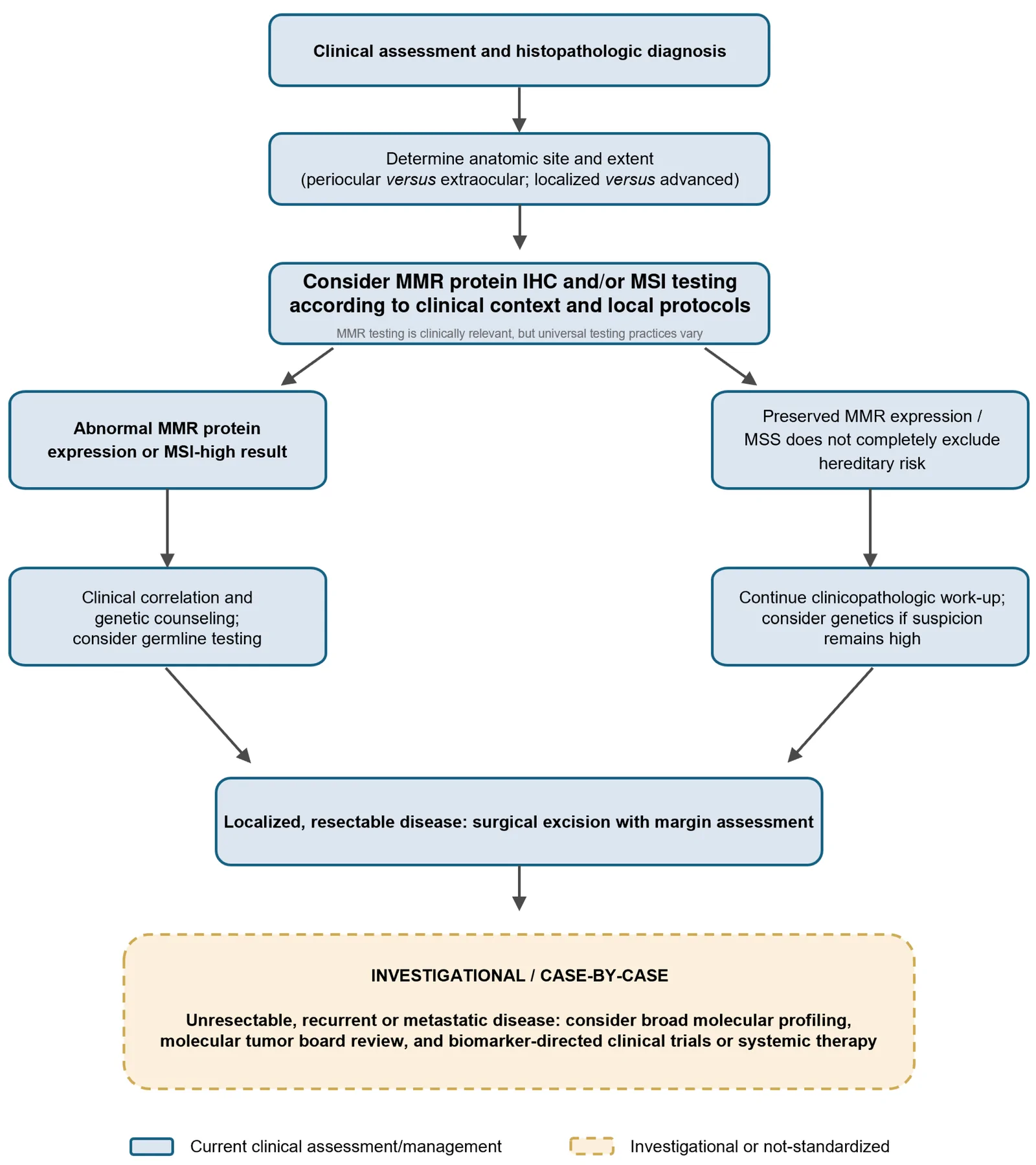
Evidence-calibrated clinical and molecular evaluation of sebaceous carcinoma. Blue boxes represent current clinicopathologic assessment or management, while the amber box identifies investigational or non-standardized approaches. MMR protein immunohistochemistry and/or MSI testing should be considered according to anatomic site, personal and family history, and local protocols; preserved MMR expression does not fully exclude hereditary risk. Broad molecular profiling and biomarker-directed systemic therapy remain individualized considerations for advanced disease and are not supported by prospective sebaceous-carcinoma-specific trials. Abbreviations: IHC, immunohistochemistry; MMR, mismatch repair; MSI, microsatellite instability; MSS, microsatellite stable.

The molecular advances reviewed above have several practical implications. Table 4 summarizes the main molecular alterations and pathways discussed throughout this review, together with their predominant anatomic context, biological interpretation, and potential clinical implications, and Figure 5 proposes a translational workflow integrating anatomic site, mismatch repair status, and molecular findings into routine clinical decision-making.

First, mismatch repair status is central. Sebaceous carcinoma, especially extraocular disease and tumors arising in patients with suggestive personal or family history, should prompt consideration of mismatch repair immunohistochemistry and/or microsatellite instability testing. Detection of mismatch repair deficiency may identify patients requiring genetic counseling for possible Lynch/Muir–Torre syndrome and may also influence systemic treatment options in advanced disease.

Second, as detailed in Section 4, anatomic site should not be assumed molecularly interchangeable: periocular tumors more often show pauci-mutational, *TP53*/*RB1*/*ZNF750*-associated biology and may also show relevant copy-number changes such as *MYC* gain. Extraocular tumors are more likely to include UV-driven and mismatch repair-deficient subsets, with greater involvement of NOTCH signaling and higher tumor mutational burden in many cases.

Third, broad molecular profiling may be considered in selected patients with unresectable, recurrent, metastatic, or treatment-refractory disease, preferably through a molecular tumor board or clinical trial. Routine broad sequencing is not established for every localized, completely excised tumor. Potentially relevant findings include dMMR/MSI-high status, high TMB, *PIK3CA* or receptor-tyrosine-kinase alterations, homologous-recombination-related alterations, and other variants with trial eligibility; their detection does not by itself establish clinical benefit from a matched therapy.

Fourth, molecular findings may refine diagnosis. In difficult histopathological cases, integration of morphology, immunohistochemistry, mismatch repair status and molecular data may help distinguish sebaceous carcinoma from benign sebaceous neoplasms and from mimics such as squamous cell carcinoma, basal cell carcinoma with sebaceous differentiation or metastatic adenocarcinoma. Transcriptomic or multi-omics classifiers may become increasingly useful in this context.

Artificial intelligence (AI) is emerging as a complementary tool for difficult differential diagnosis, and several sebaceous-carcinoma-specific retrospective studies support its potential. Luo and colleagues trained a DenseNet-161-based whole-slide model to distinguish eyelid basal cell carcinoma from sebaceous carcinoma [18]. Geng and colleagues trained a framework on 282 whole-slide images to distinguish eyelid sebaceous carcinoma from squamous cell carcinoma, validated it in an external cohort, and reported improvement in pathologist accuracy from 60.0% to 76.8% with AI assistance [19]. These results demonstrate promising performance for narrow, pairwise differentials in retrospective study settings; they do not yet establish generalizable expert-level performance in routine practice. A comprehensive diagnostic and treatment-selection algorithm would require integration of histomorphology with MMR/MSI, TMB, *TP53*/*RB1*/*ZNF750* and *MYC* status, anatomic site, prior radiation or immunosuppression, and personal/family cancer history. No such multimodal algorithm has been prospectively developed or validated specifically for sebaceous carcinoma.

Finally, the concept of sebaceous carcinoma as a molecularly heterogeneous tumor should guide research and clinical trial design. Future studies should stratify tumors by ocular versus extraocular site, mismatch repair status, tumor mutational burden, UV signature, and major driver alterations. Without such stratification, biologically distinct tumors may be inappropriately grouped, diluting clinically meaningful signals.

## 17. Future Directions

Several priorities emerge from the current literature.

Large multicenter cohorts are needed to validate molecular subgroups and prognostic biomarkers. Because sebaceous carcinoma is rare, single-center series are often underpowered, particularly for metastatic disease and treatment outcomes. International collaboration will be essential.

Integrated multi-omics should become the standard for discovery studies. Whole-genome sequencing, transcriptomics, methylation profiling, proteomics, spatial biology, and single-cell approaches can clarify how mutational processes, copy-number alterations, lineage differentiation, lipid metabolism, and immune microenvironment interact.

Molecular classifiers require clinical translation. Studies such as the multi-omics profiling of sebaceous neoplasms suggest that molecular tools may help distinguish carcinoma from benign sebaceous tumors [16]. These classifiers need validation in routine diagnostic specimens, including formalin-fixed paraffin-embedded tissue.

The immunotherapy landscape requires better definition. Mismatch repair-deficient and tumor mutational burden-high tumors are biologically plausible candidates for checkpoint blockade, but sebaceous carcinoma-specific evidence remains limited. Prospective basket trials, registry studies, and carefully annotated case series may help identify which biomarkers best predict response.

Finally, future research should distinguish tumor initiation from tumor progression. The molecular events that contribute to the initiation of a sebaceous carcinoma may not be the same as those driving pagetoid spread, nodal metastasis, distant dissemination, or resistance to therapy. Longitudinal sampling and paired primary–metastatic sequencing should be particularly valuable.

## 18. Conclusions

Sebaceous carcinoma is best understood as a molecularly heterogeneous family of sebofollicular epithelial malignancies rather than a single biological entity. Current evidence supports several major pathogenic routes: mismatch repair deficiency and microsatellite instability, ultraviolet radiation-induced mutagenesis, pauci-mutational disruption of *TP53*/*RB1*/*ZNF750*-driven cell-cycle and differentiation programs, NOTCH pathway alteration, copy-number changes including *MYC* amplification, and rarer mechanisms involving HPV, *POLE*/*POLD1*, *APOBEC* activity, base-excision repair signatures, and altered lipid metabolism.

The distinction between ocular and extraocular sebaceous carcinoma is biologically meaningful. Periocular tumors more often cluster around *TP53*/*RB1*/*ZNF750*-associated and copy-number-driven mechanisms, whereas extraocular tumors more frequently include UV-driven, mismatch repair-deficient, highly mutated, and NOTCH-altered subsets. These molecular differences have implications for diagnosis, hereditary cancer screening, prognostication, and systemic therapy.

From a translational standpoint, the most immediate priorities are identification of mismatch repair-deficient tumors, recognition of Lynch/Muir–Torre syndrome when appropriate, molecular profiling of advanced disease, and rational exploration of immune checkpoint inhibitors and targeted therapies in selected patients. As multi-omics and single-cell studies mature, sebaceous carcinoma is likely to move from a purely histopathological diagnosis toward a molecularly informed classification with direct clinical relevance.

## Figures and Tables

**Table 1:** Epidemiological, clinical, and molecular comparison between ocular/periocular and extraocular sebaceous carcinoma. Abbreviations: M:F, male-to-female ratio; MSI, microsatellite instability; SC, sebaceous carcinoma; SEER, Surveillance, Epidemiology, and End Results; UV, ultraviolet.

Feature	Ocular/Periocular SC	Extraocular SC	Key References
Anatomic origin	Eyelid/ocular adnexa, usually involving Meibomian or Zeis glands; cutaneous and/or conjunctival involvement may occur	Cutaneous sebaceous glands, most commonly head and neck	[1]
Proportion of all SC	Approximately 25–30% of cases	Approximately 70–75% of cases	[3,4]
Incidence	Disproportionately common among patients of Asian descent, accounting for approximately 40–60% of eyelid malignancies in some Asian series	Less frequent overall; population-based incidence has risen in recent decades	[1,3,5]
Sex distribution	Higher ocular: extraocular ratio in women than in men	More pronounced male predominance (extraocular M:F ≈ 1.9:1; 65.8% male, vs. 60.3% male in the overall US SEER SC cohort)	[3]
Age at diagnosis	Peak incidence 60–79 years; >98% of cases occur after age 40	Similar age distribution to ocular disease	[3]
Main risk factors	Older age, prior periocular radiotherapy, and immunosuppression; Lynch/Muir-Torre association is less characteristic than in extraocular disease	Ultraviolet exposure, Lynch/Muir-Torre syndrome, immunosuppression, and older age	[1]
Predominant molecular profile	Pauci-mutational; *TP53*/*RB1*/*ZNF750*-driven; *MYC* copy-number gain (Section 4)	More heterogeneous; enriched for UV-signature and mismatch repair-deficient/MSI tumors; higher tumor mutational burden (Section 4)	[2,5]
Typical clinical presentation	Painless nodule/thickening of the eyelid; may mimic chalazion or blepharoconjunctivitis; pagetoid spread possible	Nonspecific nodule or plaque, most often on the face; less classically recognized clinically	[1]
Main diagnostic mimics	Chalazion, cysts, blepharitis, other inflammatory eyelid conditions	Basal cell carcinoma, squamous cell carcinoma, pyogenic granuloma, sebaceous adenoma	[1]
Local recurrence	11.8%	14%	[6]
Metastasis	Approximately 12.5%	Approximately 1.8%	[6]
Survival/mortality	Higher reported disease-specific mortality in guideline cohorts (approximately 25.6%); site-based overall-survival estimates vary across population studies	Lower reported disease-specific mortality in the same guideline cohort (approximately 18%), although one SEER analysis associated extraocular site with higher all-cause mortality	[3,6]
Primary treatment	Complete surgical excision with peripheral and deep margin assessment; conjunctival mapping may assist selected periocular cases; exenteration reserved for advanced orbital disease	Complete surgical excision with peripheral and deep margin assessment; radiotherapy may be considered when surgery is unsuitable or for selected adverse features	[6]

**Table 3:** Immune and stromal features of sebaceous carcinoma: direct evidence, proposed adaptations, and evidence limitations. Abbreviations: CTLA-4, cytotoxic T-lymphocyte-associated protein 4; MMR, mismatch repair; MSI, microsatellite instability; NK, natural killer; PD-1, programmed cell death protein 1; PD-L1, programmed death-ligand 1; TMB, tumor mutational burden; Treg, regulatory T cell; UV, ultraviolet.

Context/Molecular Subset	Direct Evidence in Sebaceous Neoplasia	Potential Immune Adaptation	Evidence Level and Limitation
Carcinoma versus benign sebaceous neoplasms	Greater CD68+/CD163+ M2 macrophage and CD11c+ dendritic-cell infiltration; higher immune-cell PD-L1 in carcinoma [25]	Myeloid-rich, potentially immunosuppressive remodeling during malignant progression	Direct cross-sectional evidence; not proof of causality
Periocular/ocular-adnexal carcinoma	CD4/CD8 T cells, Treg, exhausted CD8 subsets, NK cells, B/plasma cells, dendritic cells, mast cells, fibroblasts, and endothelial cells identified by single-cell profiling [17]; compartment-specific PD-1/PD-L1/CTLA-4 associations reported [26]	Checkpoint engagement, T-cell exhaustion, regulatory T-cell activity, and stromal support	Direct but small or single-center cohorts; not validated for treatment selection
Extraocular carcinoma	Higher tumoral and immune-infiltrating PD-L1 measures and peripheral CD8-predominant infiltration reported relative to periocular tumors [20]	Adaptive checkpoint-mediated escape from an active peripheral cytotoxic response	Direct anatomic-site association; molecular subtypes were not separately analyzed
MMR-deficient/MSI	High indel burden and TMB provide a neoantigen-based rationale; immune-cell composition did not differ by MMR status in one cohort [25]	Potential increased antigenicity and checkpoint sensitivity	Treatment rationale partly extrapolated from tumor-agnostic evidence; no sebaceous-specific prospective validation
UV-driven/TMB-high	High mutation burden is established, but dedicated immune profiling of this molecular class has not been reported	Potential neoantigen enrichment and immune recognition	Biological inference only in sebaceous carcinoma
Pauci-mutational *TP53*/*RB1*/*ZNF750*	Often periocular and lower-TMB; no dedicated subtype-stratified immune profiling	Possibly greater reliance on tumor-intrinsic cell-cycle and differentiation programs	Inference only; absence of evidence is not evidence of an immune-cold phenotype
HPV-associated subset	Rare periocular subset; dedicated immune-microenvironment studies are lacking	Potential viral-antigen-driven immune recognition and immune escape	Biological inference only; no validated immune biomarker

**Table 4:** Molecular alterations and biological significance in sebaceous carcinoma. Abbreviations: AKT, protein kinase B; AR, androgen receptor; HPV, human papillomavirus; HRD, homologous recombination deficiency; MMR, mismatch repair; MSI, microsatellite instability; mTOR, mechanistic target of rapamycin; PD-L1, programmed death-ligand 1; PI3K, phosphatidylinositol 3-kinase; TMB, tumor mutational burden; UV, ultraviolet; YAP, Yes-associated protein.

Alteration/Pathway	Predominant Context	Biological Interpretation	Potential Clinical Implication
MMR deficiency/MSI	Extraocular tumors; Lynch/Muir–Torre; also sporadic/acquired cases	Defective DNA repair, indel burden, high TMB	Lynch screening; potential immunotherapy biomarker (sebaceous-carcinoma-specific clinical evidence still limited)
UV signature	Mostly extraocular, sun-exposed tumors	Actinic mutagenesis, high TMB	Possible immunotherapy relevance; overlap with keratinocyte cancer biology
*TP53*	Especially ocular/periocular; also broader	Loss of DNA damage checkpoint and genomic stability	Core driver; possible prognostic relevance
*RB1*	Especially ocular/periocular	Cell-cycle deregulation	Core driver; copy-number deletion at 13q14.3 relevant
*ZNF750*	Especially ocular/periocular	Impaired epithelial/sebofollicular differentiation	Supports differentiation-based pathogenesis
*NOTCH1*/NOTCH pathway	Extraocular and highly mutated tumors; also subset of ocular tumors	Differentiation pathway disruption; tumor suppressor role in epithelial contexts	Molecular classification; possible future target/pathway marker
PI3K/AKT/mTOR/*PIK3CA*	Reported in ocular and extraocular tumors; often ocular emphasis	Growth and survival signaling	Potential targeted therapy in selected cases
*MYC* gain/amplification	Periocular tumors	Oncogenic transcriptional program and dosage effect	Candidate driver; may support copy-number profiling
*EGFR*/*ERBB2*/tyrosine kinase alterations	Selected cases	Potential receptor-driven oncogenic signaling	Molecular tumor board consideration in advanced disease
HRD-associated genes	Selected cases	DNA repair vulnerability	Potential relevance for targeted therapy trials
HPV	Rare subset, mainly ocular adnexal	Viral oncogenesis, distinct from *TP53*/*RB1* pathway	Consider in selected younger or suggestive cases
*POLE*/*POLD1*	Rare highly mutated subset	Polymerase proofreading defect	High TMB; possible immunotherapy relevance
*AID*/*APOBEC*	Rare/signature-based subset	Cytidine deaminase-associated mutagenesis	Molecular subclassification
Cholesterol metabolism/*SCARB1*/*PPARG*	Eyelid sebaceous carcinoma transcriptomic studies	Altered lipid handling and sebaceous differentiation	Candidate biomarker/biological vulnerability
PD-L1/immune microenvironment	Variable; potentially enriched in immunogenic subsets	Immune evasion and checkpoint biology	Supports immune checkpoint inhibitor rationale
YAP/Hippo pathway	Eyelid sebaceous carcinoma	Context-dependent growth/differentiation regulation	Candidate prognostic marker for local recurrence

## Data Availability

No new data were created or analyzed in this study. Data sharing is not applicable to this article.

## Abbreviations

The following abbreviations are used in this manuscript:

AI	artificial intelligence
AID	activation-induced cytidine deaminase
AKT	protein kinase B
APOBEC	apolipoprotein B mRNA-editing enzyme catalytic polypeptide-like
AR	androgen receptor
CAF	cancer-associated fibroblast
COSMIC	Catalogue of Somatic Mutations in Cancer
CTLA-4	cytotoxic T-lymphocyte-associated protein 4
dMMR	mismatch repair-deficient
HPV	human papillomavirus
HRD	homologous recombination deficiency
IHC	immunohistochemistry
MMR	mismatch repair
MSI	microsatellite instability
MSS	microsatellite stable
MTS	Muir–Torre syndrome
mTOR	mechanistic target of rapamycin
NGS	next-generation sequencing
NK	natural killer
PD-1	programmed cell death protein 1
PD-L1	programmed death-ligand 1
PI3K	phosphatidylinositol 3-kinase
SC	sebaceous carcinoma
SC-E	extraocular sebaceous carcinoma
SC-O	ocular/periocular sebaceous carcinoma
SEER	Surveillance, Epidemiology, and End Results
TMB	tumor mutational burden
TME	tumor microenvironment
Treg	regulatory T cell
UV	ultraviolet
WES	whole-exome sequencing
WGS	whole-genome sequencing
WTS	whole-transcriptome sequencing
YAP	Yes-associated protein
